# Implementation of the FL-REACH Caregiver Intervention: Translation in an Outpatient Memory Disorders Clinic

**DOI:** 10.1093/geroni/igaa057.1544

**Published:** 2020-12-16

**Authors:** Tracy Wharton, Daniel Paulson, Nicholas James, Rosemary Laird, Barbara Mendez Campos, Gayle Shepherd

**Affiliations:** 1 University of Central Florida, Orlando, Florida, United States; 2 Advent Health, Orlando, Florida, United States

## Abstract

The REACH II intervention is the gold-standard in dementia caregiver interventions. The FL-REACH translation is a novel adaptation offered in both English and Spanish to an outpatient memory disorder clinic at an urban, Southeastern healthcare system. This pre-post feasibility trial involves 6 sessions (4 in person at the clinic and 2 by phone) with the identified caregiver and any other family who wish to attend, which may also include the person living with dementia. The program is focused on early stage post-diagnosis, and is structured around building rapport, empowering families to build support networks, and teaching skills and knowledge-based material. Twenty four of the 60-participant target sample have consented to participate in this ongoing study. Change on the Preparedness for Caregiving Scale is significant (t=3.03, p=.001, Cohen’s d=2.49). Means for the Zarit Burden 12-item scale went from 24.5 to 13.17 (t=-6.65, p=.03, Cohen’s d=3.53). Access by care recipients to dangerous objects decreased (67% to 14%). Confidence in ability to use behavioral strategies in caregiving increased from 8% at baseline to 72% at study completion. Satisfaction surveys indicate high satisfaction with all elements of the intervention. These outcomes are consistent with existing data regarding utility of the REACH framework and reflect feasibility of delivering an adapted program model in an outpatient clinic environment. A future randomized controlled trial should examine whether early intervention and training reduces utilization of emergency care over time and improves quality of life for families.

